# Early gut microbial and metabolic dysregulation with subclinical cardiac alterations in a nonhuman primate model of Rett syndrome

**DOI:** 10.1002/imt2.70137

**Published:** 2026-06-09

**Authors:** Ting Zhang, Xiaopeng Wang, Peng Li, Junyu Zhang, Wenjie Sun, Zhen Zhang, Yan Zhuo, Wenting Guo, Yongchang Chen

**Affiliations:** ^1^ State Key Laboratory of Primate Biomedical Research, Institute of Primate Translational Medicine Kunming University of Science and Technology Kunming China; ^2^ Faculty of Life Science and Technology Kunming University of Science and Technology Kunming China; ^3^ Yunnan Key Laboratory of Primate Biomedical Research Kunming China; ^4^ Southwest United Graduate School Kunming China

## Abstract

Longitudinal multi‐omics profiling of a nonhuman primate Rett syndrome (RTT) model reveals early systemic alterations. RTT monkeys exhibited postnatal growth retardation, intestinal structural abnormalities, and low‐grade systemic inflammation. Gut microbiome analysis showed delayed microbial maturation and age‐discordant dysbiosis, including altered *Firmicutes/Bacteroidetes* ratios and persistent community restructuring. Fecal metabolomics revealed reduced short‐chain fatty acids (SCFAs), disrupted microbe–metabolite networks, and broad alterations in lipid, amino acid, and energy metabolism. Electrocardiogram (ECG) identified prolonged corrected QT interval (QTc) and subclinical cardiac electrophysiological changes. Integrated multi‐omics analyses indicate that RTT involves early, coordinated dysregulation across gut microbial, metabolic, immune, and peripheral physiological systems, supporting its characterization as a systemic disorder from the early postnatal stage.

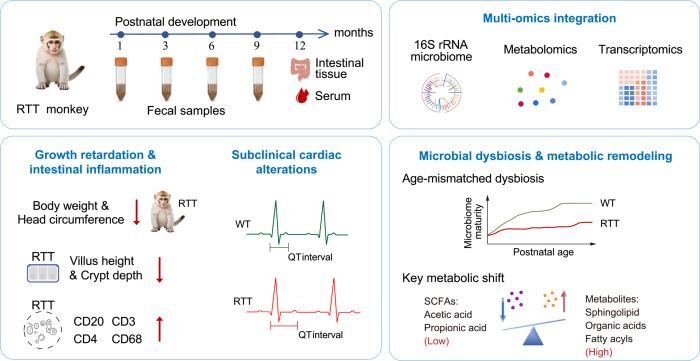

To the editor,

Rett syndrome (RTT) is a severe X‐linked neurodevelopmental disorder caused primarily by mutations in the *MECP2* gene. In addition to its well‐recognized neurological manifestations, RTT is frequently accompanied by systemic abnormalities, including gastrointestinal dysfunction, growth impairment, immune dysregulation, and cardiovascular complications [1, 2, 3]. Clinical symptoms typically emerge between 6 and 18 months of age, including developmental slowing, loss of acquired skills, and autonomic abnormalities [2, 3], but it remains unclear whether peripheral alterations also arise during this early developmental window. To address this question, we performed longitudinal phenotypic and multi‐omics profiling in a MeCP2‐mutant monkey model of RTT from birth through early postnatal development, with the aim of determining whether early gut microbial, metabolic, and peripheral organ abnormalities are already present during postnatal development in a physiologically relevant primate model.

## EARLY SYSTEMIC AND INTESTINAL ABNORMALITIES IN RTT MONKEYS

**Figure 1:**
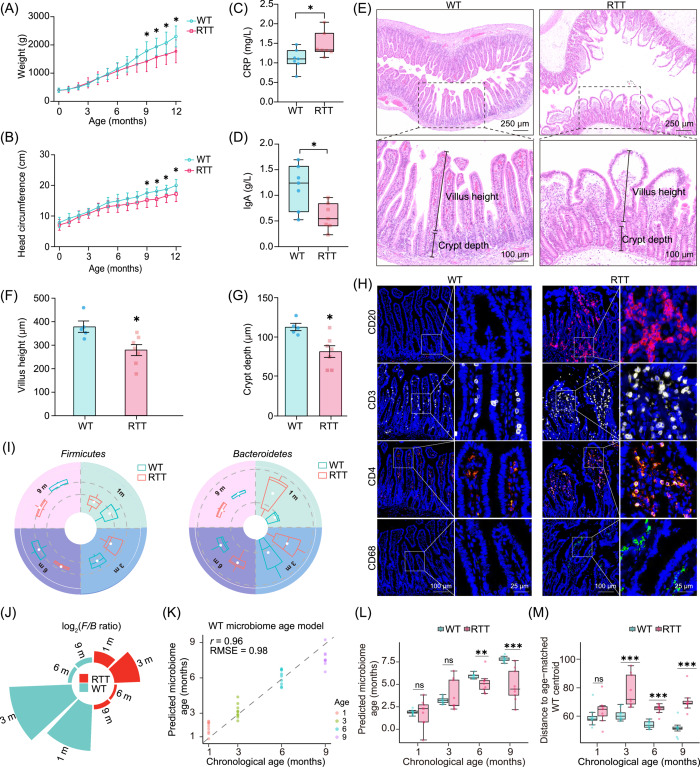
Early systemic, intestinal, and gut microbial abnormalities in Rett syndrome (RTT) monkeys. Longitudinal measurements of body weight (A) and head circumference (B) in wild‐type (WT) and RTT monkeys from birth to 12 months of age. Serum levels of C‐reactive protein (CRP) (C) and immunoglobulin A (IgA) (D) in WT and RTT monkeys at 12 months of age. (E) Representative hematoxylin and eosin staining (H&E) of small intestinal tissues from WT and RTT monkeys at 12 months of age. Upper panels show low‐magnification views, and lower panels show enlarged regions illustrating villus height and crypt depth. Scale bars, 250 μm (upper panels) and 100 μm (lower panels). Quantification of villus height (F) and crypt depth (G) in small intestinal sections from WT and RTT monkeys at 12 months of age. (H) Representative multiplex immunofluorescence staining of small intestinal tissues showing CD20 (pink), CD3 (white), CD4 (orange), and CD68 (green) signals in WT and RTT monkeys at 12 months of age. Nuclei were counterstained with DAPI (blue). Scale bars, 100 μm (low magnification) and 25 μm (high magnification). (I) Relative abundances of the major phyla *Firmicutes* and *Bacteroidetes* in WT and RTT monkeys at 1, 3, 6, and 9 months of age. (J) Log_2_‐transformed *Firmicutes/Bacteroidetes* (*F/B*) ratio across development in WT and RTT monkeys. (K) WT microbiome age model performance evaluated by leave‐one‐animal‐out cross‐validation, showing the relationship between predicted microbiome age and chronological age. (L) Predicted microbiome age in WT and RTT monkeys across development. (M) Age‐matched compositional deviation of WT and RTT samples relative to the age‐matched WT microbiota centroid. For panels (A)–(B), *n* = 9 biologically independent monkeys per group. For panels (C)–(D), *n* = 7 biologically independent monkeys per group. For panels F and G, *n* = 7 for RTT and *n* = 5 for WT. Data are presented as mean ± SEM for panels (A), (B), (F), and (G). Statistical significance for panels (A) and (B) was assessed using two‐way repeated‐measures ANOVA. Statistical significance for panels (C), (D), (F), (G), (L), and (M) was assessed using the Wilcoxon rank‐sum test. For panels (I), (J), (L), and (M), RTT and WT monkeys were compared separately at each time point to assess group differences at specific developmental stages. ns, not significant; *p < 0.05; **p < 0.01; ***p < 0.001.

We longitudinally followed nine RTT monkeys and nine age‐matched wild‐type (WT) controls. RTT monkeys showed progressive somatic impairment during development. Body weight and head circumference became significantly reduced at later stages, indicating emerging growth retardation (Figure 1A,B). These phenotypic changes were accompanied by elevated serum C‐reactive protein and reduced immunoglobulin A (IgA) (Figure 1C,D), suggesting low‐grade systemic inflammation and altered mucosal immune status [4].

We next examined intestinal pathology. Histological analysis of the small intestine revealed reduced villus height, decreased crypt depth, and inflammatory changes in RTT monkeys (Figure 1E−G). To strengthen the evidence for intestinal immune remodeling, we additionally assessed immune‐cell markers and observed increased CD20‐, CD3‐, CD4‐, and CD68‐positive cells in RTT intestinal tissue (Figure 1H). Together, these data support the presence of early peripheral abnormalities in RTT monkeys involving growth, mucosal integrity, and immune regulation, indicating that disease‐associated dysfunction extends beyond the nervous system at early postnatal stages [5, 6, 7].

## DELAYED MICROBIOME MATURATION AND AGE‐MISMATCHED DYSBIOSIS

To examine gut microbial development, we performed longitudinal 16S rRNA profiling of fecal samples collected at 1, 3, 6, and 9 months of age. Sequencing depth was sufficient across samples (Figure S1A), and both groups showed age‐related increases in richness (Figure S1B), consistent with postnatal microbiome maturation. However, RTT monkeys progressively diverged from WT controls over time. Alpha diversity was comparable between groups at 1 and 3 months, but RTT monkeys failed to show the normal age‐associated increase in diversity and richness at later stages (Figure S1C). Beta diversity analyses likewise demonstrated progressive separation between RTT and WT communities, indicating altered developmental trajectories of the gut microbiota in RTT (Figure S1D). These observations are consistent with previous reports linking RTT to gut microbial alterations and broader host‐microbiota dysregulation in neurodevelopmental disorders [8, 9, 10].

At the phylum level, both groups were dominated by *Firmicutes* and *Bacteroidetes* (Figure S1E), but RTT monkeys displayed a trend toward a lower *Firmicutes/Bacteroidetes* (*F/B*) ratio driven by lower *Firmicutes* and relative expansion of *Bacteroidetes* (Figure 1I,J). At the genus level, *Prevotella*, *Bifidobacterium*, *Faecalibacterium*, *Blautia*, *Lactobacillus*, *Bacteroides*, and *Holdemanella* remained dominant (Figure S1F), and several genera also showed stage‐dependent alterations, including increased *Bacteroides* at earlier stages (1 and 3 months) and increased *Bifidobacterium* at later stages (6 and 9 months), whereas taxa such as *Blautia* were reduced at 3 months (Figure S1G–I). LEfSe analysis further identified RTT‐enriched taxa including *Helicobacter, Collinsella, Lactobacillus, Enterococcus*, and *Clostridioides* (Figure S1J,K), supporting persistent restructuring of the microbial ecosystem.

A key question was whether these microbial changes simply reflected generalized developmental delay. To address this, we performed a microbiome‐age analysis using the WT cohort as a developmental reference. The WT‐based model accurately predicted chronological age in leave‐one‐animal‐out validation, supporting its use for assessing microbiome maturation (*r* = 0.96, RMSE = 0.98, Figure 1K). When applied to RTT monkeys, predicted microbiome age was comparable to WT at 1 and 3 months but significantly lower at 6 and 9 months, indicating delayed microbial maturation at later stages (Figure 1L). However, age‐matched compositional deviation analysis showed that RTT samples were already significantly more distant from the WT centroid at 3 months and remained divergent thereafter (Figure 1M). Thus, the RTT microbiome is not explained solely by a younger WT‐like state. Instead, RTT monkeys exhibit both delayed microbiome maturation and distinct age‐matched compositional dysbiosis.

## SCFAS DEPLETION AND BROADER METABOLIC REMODELING

**Figure 2:**
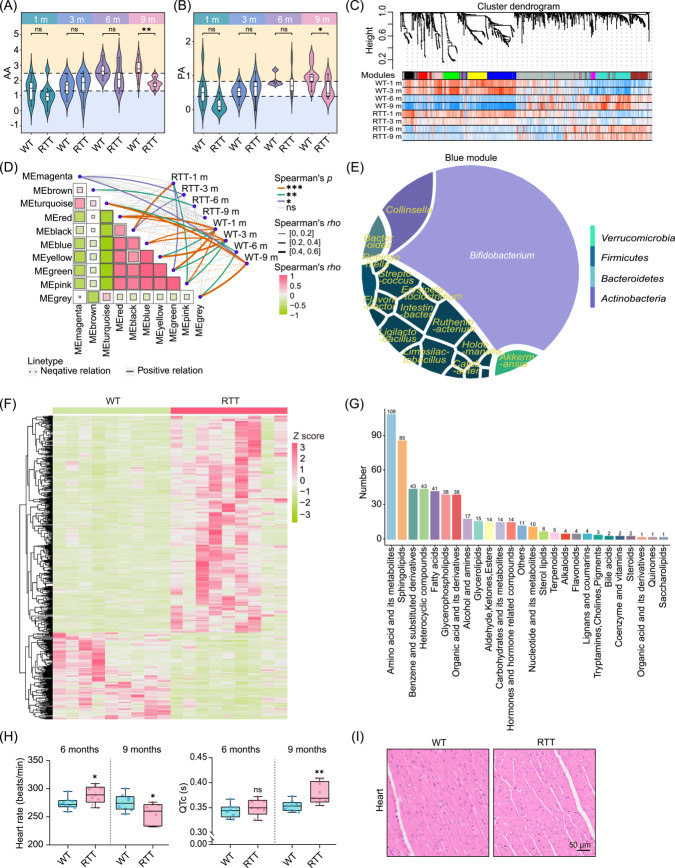
Short‐chain fatty acids (SCFAs) disruption, metabolomic remodeling, and subclinical cardiac alterations in RTT monkeys. (A, B) Fecal concentrations of acetic acid (AA, A) and propionic acid (PA, B) in WT and RTT monkeys at 1, 3, 6, and 9 months of age. (C) Weighted gene co‐expression network analysis (WGCNA) dendrogram of microbial amplicon sequence variants (ASVs), showing module assignment. (D) Correlation analysis between microbial modules and developmental groups in WT and RTT monkeys, with results indicating age‐dependent remodeling of SCFAs‐associated microbial networks. (E) Composition of the MEblue module, which contained all seven SCFAs and was centered on *Bifidobacterium* as a major hub genus. (F) Heatmap of differentially abundant fecal metabolites in WT and RTT monkeys at 9 months of age. (G) Classification of differentially abundant metabolites by chemical category. (H) Longitudinal electrocardiogram (ECG)‐derived heart rate and corrected QT interval (QTc) in WT and RTT monkeys at 6 and 9 months of age. (I) Representative H&E staining of cardiac tissue from WT and RTT monkeys at 1 year of age. Scale bar, 50 μm. For panels A, B, F, G, and H, *n* = 9 biologically independent monkeys per group. For panels A, B, and H, statistical significance was assessed using the Wilcoxon rank‐sum test. RTT and WT monkeys were compared separately at each time point to assess group differences at specific developmental stages. ns, not significant; *p < 0.05; **p < 0.01.

Because taxonomic dysbiosis often has functional consequences, we quantified seven fecal short‐chain fatty acids (SCFAs) across development (Tables S1 and S2). RTT monkeys exhibited reduced levels of acetic acid (AA) and propionic acid (PA) at 9 months of age (Figure 2A,B). We then examined microbiota‐SCFAs relationships using network‐based analyses. Network analysis integrating amplicon sequence variants abundance and SCFAs concentrations identified nine co‐expression modules (Figure 2C). In WT monkeys, SCFAs were embedded within an organized microbial co‐abundance network, whereas in RTT monkeys, these relationships weakened markedly over time. By 9 months, the RTT group no longer showed stable module‐level associations with SCFAs (Figure 2D), suggesting disruption of the core microbial metabolic network. The MEblue module, which contained all seven SCFAs, was enriched for members *Actinobacteria*, *Bacteroidetes*, *Firmicutes*, and *Verrucomicrobia*, with *Bifidobacterium* emerging as a central hub genus (Figure 2E).

This loss of network stability was further reflected in microbial‐SCFAs coupling. In RTT monkeys, the number of significant genus‐SCFAs associations fell by approximately 60%, and the correlation range narrowed markedly, consistent with reduced network complexity. Notably, about 61% of genera displayed reversed SCFAs associations in RTT, including shifts from negative to positive correlations for *Bifidobacterium*, *Eubacterium*, *Blautia*, *Dorea*, and *Dialister*, and opposite shifts for *Faecalibacterium*, *Phocaeicola*, and *Coprococcus* (Figure S2A). Such reversals suggest functional rewiring of microbial cross‐feeding relationships rather than a purely taxonomic disturbance, potentially reflecting altered substrate flow, reduced SCFAs availability, and changed luminal conditions in the inflamed RTT intestine [11, 12, 13, 14].

To assess broader downstream consequences, we performed fecal metabolomics at 9 months of age. A total of 3,595 metabolites were detected, of which 526 differed significantly between RTT and WT groups (Tables S3 and S4). Multivariate analysis showed clear separation between RTT and WT groups (Figures 2F and S2B,C), indicating robust metabolic divergence. Differential metabolites were enriched in amino acids and derivatives, sphingolipids, phenyl compounds, organic acids, and fatty acyls (Figure 2G), pointing to broad disturbances in energy and lipid metabolism. Pathway analysis highlighted alterations in caffeine metabolism, sphingolipid metabolism, ether lipid metabolism, ascorbate and aldarate metabolism, and aromatic amino‐acid biosynthesis, among others (Figure S2D). Because annotation confidence varies in metabolomics, these results are best interpreted as metabolically relevant pathway‐level shifts rather than definitive evidence for single‐pathway activation.

Integrated microbiota‐metabolite analysis further showed that RTT was associated with a simplified and fragmented interaction network, with fewer metabolite associations per genus, lower node centrality, and a 40%−50% reduction in modularity relative to WT monkeys. Several genera, including *Dorea, Blautia, Coprococcus*, and *Intestinibacter*, that occupied central positions in WT monkeys lost connectivity in RTT, whereas other taxa, *Bifidobacterium, Collinsella, Negativibacillus*, and *Ligilactobacillus*, became more central under disease conditions, indicating large‐scale rewiring of microbiome‐metabolite relationships (Figure S2E).

Integration of transcriptomic and metabolomic data further identified highly connected molecular hubs, including d‐glucose, glycine, oleic acid, dodecanoic acid, palmitic acid, l‐aspartic acid, arachidonic acid, ornithine, theophylline, and adenine (Figure S2F). Joint pathway analysis highlighted glycerophospholipid metabolism, glycerolipid metabolism, sphingolipid metabolism, and peroxisome proliferator‐activated receptor signaling as the most prominently perturbed pathways (Figure S2G). These pathways are closely linked to membrane composition, energy homeostasis, inflammatory regulation, and cardiovascular function, suggesting that RTT‐associated gut dysbiosis is accompanied by broader lipid and immune‐metabolic disturbances [11, 12, 13, 15].

Together, these data show that RTT is associated with coordinated disruption of microbial ecology, SCFAs production, and metabolic homeostasis during early development.

## SUBCLINICAL CARDIAC ALTERATIONS WITH CAUTIOUS INTERPRETATION OF EXPLORATORY ANALYSES

Electrocardiogram (ECG) assessment revealed altered heart rate at 6 and 9 months and prolonged corrected QT interval (QTc) at 9 months in RTT monkeys (Figure 2H), providing direct evidence of early cardiac electrophysiological abnormalities. In contrast, serum Troponin‐I and B‐type natriuretic peptide levels were not significantly changed at 1 year of age (Table S5), and cardiac hematoxylin and eosin staining revealed no overt histopathological abnormalities at the same age (Figure 2I). These findings therefore support the presence of subclinical cardiac alterations, rather than overt myocardial injury or structural cardiomyopathy.

To place these physiological findings in a broader systems context, we further performed exploratory analyses of microbial and host pathway annotations. PICRUSt2‐based Kyoto Encyclopedia of Genes and Genomes analysis suggested relative enrichment of gene families linked to arachidonic acid metabolism, inflammatory processes, and conventionally disease‐labeled categories, together with arrhythmogenic right ventricular cardiomyopathy (ARVC)‐ and dilated cardiomyopathy (DCM)‐related annotations (Figure S3). Because these categories largely reflect conserved functional modules rather than direct disease states, they are interpreted here only as shifts in predicted microbial functional potential. Consistently, bulk RNA‐seq of small intestinal tissue identified broad transcriptional alterations in RTT monkeys and enrichment of ARVC‐ and DCM‐related annotations in intestinal tissue (Figure S4). Taken together, these results suggest broader systemic molecular perturbation with possible cardiovascular relevance, but they do not constitute evidence of overt cardiac pathology.

A more systematic prioritization of RTT‐associated microbial features was obtained across 72 fecal samples using 15 machine learning algorithms and 279 model combinations. A neural network‐based multilayer perceptron showed the best overall performance, with an external test area under the curve (AUC) of 0.825 (Figure S5). Consensus feature aggregation identified recurrent RTT‐associated taxa, including *Blautia, Flintibacter, Negativibacillus, Bacteroides, Intestinibacter, Phocaeicola, Collinsella, Dorea, Ruthenibacterium*, and *Eubacterium* (Figure S6A), whereas integration with neural‐network ranking yielded 18 RTT‐associated genera overall, with *Prevotella* and *Bifidobacterium* among the most abundant in RTT monkeys (Figure S6B,C). Exploratory Mendelian randomization (MR) using public human genome‐wide association study summary statistics [16] suggested nominal associations between several of these taxa and arrhythmia‐ or cardiomyopathy‐related traits (Figure S7A). Among them, *Dorea* was recurrently prioritized across analytical approaches (Figure S7B), suggesting that it may represent a relatively robust microbial feature of RTT‐associated dysbiosis. However, given the limitations of microbiome‐based MR and the cross‐species nature of the analysis, any relationship between these taxa and cardiac phenotypes should be regarded as hypothesis‐generating rather than causal.

## STUDY LIMITATIONS

Our study has several limitations. Some analyses remain constrained by sample size, and more detailed cardiac phenotyping will be needed to define the nature of cardiac involvement more fully. In addition, whether modulation of specific microbial or metabolic pathways can influence peripheral or cardiac phenotypes in RTT remains unknown. Functional annotations derived from 16S data remain predictive, metabolomics pathway assignments depend on annotation confidence, and the transcriptomic and MR analyses do not establish causality. The machine‐learning analysis should also be interpreted cautiously, as the longitudinal dataset was split at the sample level rather than the subject level, which may have introduced optimistic bias. Nevertheless, the convergence of longitudinal microbiota, SCFAs, metabolomic, histological, and host transcriptomic evidence supports the robustness of the overall biological pattern and suggests that systemic gut microbial and metabolic dysregulation is an early feature of RTT in this primate model [17, 18, 19].

In summary, longitudinal analysis of a primate RTT model reveals early and progressive dysregulation of gut microbial development, SCFAs metabolism, and broader fecal metabolic homeostasis, together with intestinal inflammatory changes and subclinical cardiac electrophysiological abnormalities. These findings support the view that RTT is an early systemic disorder with coordinated gut, metabolic, immune, and peripheral physiological alterations. By defining this early postnatal trajectory in a nonhuman primate model, our study provides a framework for future mechanistic and therapeutic studies targeting microbiome‐ and metabolism‐related pathways in RTT.

## AUTHOR CONTRIBUTIONS

**Ting Zhang**: Writing—original draft; investigation. **Xiaopeng Wang**: data curation; formal analysis; methodology. **Peng Li**: Investigation; writing—original draft. **Junyu Zhang**: Methodology; formal analysis; data curation. **Wenjie Sun**: Investigation. **Zhen Zhang**: Investigation. **Yan Zhuo**: Investigation. **Wenting Guo**: Writing—review and editing; investigation. **Yongchang Chen**: Resources; supervision; funding acquisition; writing—review and editing; project administration.

## CONFLICT OF INTEREST STATEMENT

The authors declare no conflicts of interest.

## ETHICS STATEMENT

All animal experiments in this study were reviewed and approved by the Animal Ethics Committee of Kunming University of Science and Technology (Approval No. KUST202301024).

## Supporting information


**Figure S1:** Longitudinal profiling of gut microbial diversity and community structure in RTT monkeys.
**Figure S2:** Fecal metabolomic alterations and microbiota‐metabolite network remodeling in RTT monkeys.
**Figure S3:** Altered predicted microbial functional potential in RTT monkeys across development.
**Figure S4:** Transcriptomic alterations in small intestinal tissues from WT and RTT monkeys.
**Figure S5:** Performance evaluation of machine learning models for identifying RTT associated microbial genera.
**Figure S6:** Prioritization of RTT‐associated microbial features by machine‐learning analysis.
**Figure S7:** Exploratory Mendelian randomization analysis of RTT‐associated microbial genera and cardiovascular‐related traits.

**Table S1:** Linear regression equations and coefficients of determination (*R* ^2^) for short‐chain fatty acids (SCFAs) quantification by GC‐MS/MS.
**Table S2:** SCFAs levels in WT and RTT monkeys at 1, 3, 6, and 9 months of age.
**Table S3:** Fecal metabolites detected in WT and RTT monkeys at 9 months of age.
**Table S4:** Differentially abundant fecal metabolites in WT and RTT monkeys at 9 months of age.
**Table S5:** Serum Troponin‐I and B‐type natriuretic peptid (BNP) measurements at 1 year of age.

## Data Availability

All the sequencing data have been deposited in the Genome Sequence Archive at the National Genomics Data Center under project accession number PRJCA053030 (https://ngdc.cncb.ac.cn/bioproject/browse/PRJCA053030). The datasets include bulk RNA‐seq data (CRA035002), 16S rRNA gene sequencing data (CRA035000), and SCFAs and metabolomics data (OMIX013525). The figure‐related scripts and data were deposited in https://github.com/zjy720129-web/Zhangting2026iMeta/tree/main. Supplementary materials (methods, figures, tables, graphical abstract, slides, videos, Chinese translated version, and updated materials) may be found in the online DOI or iMeta Science http://www.imeta.science/.
