# Supplementary material for: Early gut microbial and metabolic dysregulation with subclinical cardiac alterations in a nonhuman primate model of Rett syndrome
Source: Imeta. 2026 Jun 9;5(3):e70137. doi: 10.1002/imt2.70137 (PMC13377406; doi:10.1002/imt2.70137)
Supplement: Supplementary file 1 — Figure S1: Longitudinal profiling of gut microbial diversity and community structure in RTT monkeys. Figure S2: Fecal metabolomic alterations and microbiota‐metabolite network remodeling in RTT monkeys. Figure S3: Altered predicted microbial functional potential in RTT monkeys across development. Figure S4: Transcriptomic alterations in small intestinal tissues from WT and RTT monkeys. Figure S5: Performance evaluation of machine learning models for identifying RTT associated microbial genera. Figure S6: Prioritization of RTT‐associated microbial features by machine‐learning analysis. Figure S7: Exploratory Mendelian randomization analysis of RTT‐associated microbial genera and cardiovascular‐related traits. [file IMT2-5-e70137-s002.docx]

**Supporting information to**

**Early gut microbial and metabolic dysregulation with subclinical cardiac alterations in a nonhuman primate model of Rett syndrome**

**Running title**: Early gut microbial and metabolic dysregulation in a nonhuman primate Rett syndrome model

Ting Zhang^1,2,3#^, Xiaopeng Wang^1,4#^, Peng Li^1#^, Junyu Zhang^1,4#^, Wenjie Sun^1,3^, Zhen Zhang^1^, Yan Zhuo^1,3^, Wenting Guo^1,3^*, Yongchang Chen^1,2,3,4^*

^1^State Key Laboratory of Primate Biomedical Research, Institute of Primate Translational Medicine, Kunming University of Science and Technology, Kunming 650500, China

^2^Faculty of Life Science and Technology, Kunming University of Science and Technology, Kunming 650500, China

^3^Yunnan Key Laboratory of Primate Biomedical Research, Kunming 650500, China

^4^Southwest United Graduate School, Kunming 650092, China

^#^These authors contributed equally: Ting Zhang, Xiaopeng Wang, Peng Li, Junyu Zhang

^*^Correspondence: [guowt@lpbr.cn](mailto:guowt@lpbr.cn) (Wenting Guo) and [chenyc@lpbr.cn](mailto:chenyc@lpbr.cn) (Yongchang Chen)

**ORCID ID**

Yongchang Chen ID: 0000-0002-3856-3576

Wenting Guo ID: 0000-0002-1370-7764

**Supplementary Materials**

**MATERIALS AND METHODS**

**Animals and Sample Collection**

A total of nine Rett syndrome (RTT) monkeys and nine age-matched wild-type (WT) monkeys were used in this study. All animals were housed under controlled temperature and humidity conditions and maintained under standardized husbandry protocols. Fecal samples were collected in the morning before scheduled feeding. Because feeding frequency and diet composition differed across developmental stages, an age-adapted pre-feeding interval was used: approximately 4 hours for monkeys aged 3, 6, and 9 months, and approximately 3 hours for 1-month-old infants that were still mainly fed liquid formula. This procedure was applied consistently within each age group under comparable morning husbandry conditions. Monkeys were artificially fed, and no antibiotics, probiotics, or prebiotics were used during sample collection.

Fresh fecal samples were collected every three months, immediately transferred into sterile cryovials, and stored at −80 °C until analysis. Growth parameters, including body weight, head circumference, and body length, were monitored throughout the study. None of the animals received antibiotics, probiotics, or prebiotics during the sampling period. All experimental procedures were approved by the Animal Ethics Committee of Kunming University of Science and Technology (Approval No. KUST202301024).

**Serum CRP, IgA, Troponin-I and BNP Measurement**

Venous blood (0.8 mL) was collected from one year old RTT and WT monkeys in the early morning after overnight fasting. After standing at room temperature for 30 min, samples were centrifuged at 1200 × g for 10 min to obtain serum. Serum C-reactive protein (CRP) and immunoglobulin A (IgA) levels were measured by immunoturbidimetric assay using commercial reagent kits on a fully automated biochemical analyzer (Mindray BS-2000M). Serum troponin-I was measured using a chemiluminescence immunoassay analyzer (Mindray CL-6000i), and B-type natriuretic peptid (BNP) was measured on an Antu A2000 Plus analyzer. All measurements were performed strictly according to the manufacturer’s instructions.

**Hematoxylin and Eosin (H&E) Staining**

Small intestinal tissues were fixed, dehydrated, embedded in OCT, and sectioned at 10 μm. Sections were stained using a commercial H&E staining kit (Solarbio, #G1120) according to the manufacturer’s instructions. Images were acquired using a panoramic digital slide scanner (SLIDEVIEW VS200, Olympus, Germany).

**Multiplex immunofluorescence staining**

Paraffin-embedded small intestinal sections were deparaffinized, rehydrated, and subjected to antigen retrieval by microwave heating. Multiplex immunofluorescence staining was performed using a five-color multiplex immunofluorescence kit (AFIHC027, AiFang Biological, China) according to the manufacturer’s instructions. Endogenous peroxidase activity was blocked with 3% hydrogen peroxide for 15 min, followed by blocking with 10% goat serum for 15 min at room temperature.

Sections were sequentially incubated with primary antibodies overnight at 4 °C, followed by incubation with a Polymer-HRP anti-mouse/rabbit universal secondary antibody (AFIHC001, AiFang Biological, China) for 30 min at room temperature and TYR fluorophore development for 8 min. Microwave stripping was performed between staining cycles. Primary antibodies included CD3 (ab16669, 1:2000, Abcam), CD4 (AFRM0053, 1:2000, AiFang Biological), CD20 (AF20012, 1:2000, AiFang Biological), and CD68 (AF20022, 1:4000, AiFang Biological). Nuclei were counterstained with DAPI, and images were acquired using an eight-channel fluorescence digital slide scanner (AF-KL-20-8, AiFang Biological, China).

**Microbiota 16S rRNA Gene Sequencing**

Microbial genomic DNA was extracted from approximately 250 mg of each fecal sample using the cetyltrimethylammonium bromide (CTAB) method (Nobleryder, China) according to the manufacturer’s instructions. DNA concentration and purity were assessed using a NanoDrop spectrophotometer (Thermo Fisher Scientific, USA), and DNA was diluted to 1 ng/μL for PCR amplification. The bacterial 16S rRNA gene V3-V4 region was amplified using fusion primers (forward, 5′-CTAYGGGRBGCASCAG-3′; reverse, 5′-GGACTACNNGGGTATCTAAT-3′). Amplicons were purified using the MinElute PCR Purification Kit (Qiagen, Germany). Sequencing libraries were prepared using the TruSeq DNA PCR-Free Sample Preparation Kit (Illumina, San Diego, CA, USA), quality-checked using a Qubit fluorometer, an Agilent 2100 Bioanalyzer, and quantitative PCR, and sequenced on the Illumina NovaSeq 6000 platform (Illumina, San Diego, CA, USA).

**Short-Chain Fatty Acids Quantification**

Short-chain fatty acids (SCFAs), including acetic, propionic, isobutyric, butyric, isovaleric, valeric, and hexanoic acids, were quantified by GC-MS/MS. Briefly, approximately 20 mg of fecal sample was weighed and extracted with 1 mL of 0.5% phosphoric acid. After homogenization, ultrasonication, and centrifugation, 100 μL of the supernatant was mixed with 500 μL of methyl tert-butyl ether containing 2-methylpentanoic acid as the internal standard. The organic phase was analyzed on an Agilent 7890B gas chromatograph coupled to an Agilent 7000D mass spectrometer equipped with a DB-FFAP capillary column (30 m × 0.25 mm × 0.25 μm; J&W Scientific, USA). Helium was used as the carrier gas at 1.2 mL/min, and data were acquired in multiple reaction monitoring mode.

Absolute quantification was performed using internal standard-based calibration. Separate calibration curves were generated for each analyte over a concentration range of 0.005−20 μg/mL, and quantification was based on analyte-to-internal-standard peak area ratios. SCFAs concentrations were calculated as mg/g feces according to the corresponding calibration equations, extraction volume, and sample weight. For each analyte, the regression equation, coefficient of determination (*R²*), weighting factor (1/x), and lower and upper limits of quantification were determined. All analytes showed good linearity (*R²* > 0.998). The corresponding regression equations and coefficients of determination are listed in Table S1.

**Metabolomic Profiling**

Fecal metabolomic profiling was performed using a combined untargeted and targeted LC-MS/MS workflow to maximize metabolite coverage. Hydrophilic and hydrophobic metabolites were extracted separately and analyzed under complementary chromatographic conditions. Untargeted metabolite detection and annotation were first conducted on a UPLC-QTOF-MS/MS platform using pooled quality-control samples, and the resulting annotated metabolite information was subsequently used to generate a project-specific library for relative quantification of all samples on a quadrupole linear ion trap (QTRAP) platform in multiple reaction monitoring (MRM) mode. Peak extraction, integration, and correction were performed using Analyst software (version 1.6.3), and metabolite abundances were represented by integrated peak areas.

Metabolite annotation was based on multiple orthogonal criteria, including precursor ion mass, MS/MS fragmentation spectra, and retention time (RT). According to the vendor report, untargeted annotations were assigned using an in-house reference database (MWDB) containing experimentally acquired MS/MS spectra and RT information, as well as an integrated public database, DB-all, which including human metabolome database (HMDB), Metlin, and Kyoto Encyclopedia of Genes and Genomes (KEGG). Supplementary AI-predicted and MetDNA-assisted annotation strategies were also applied. Authentic chemical standards (> 98% purity) were available in the reference system and were used for structural validation where applicable.

To align annotation confidence with the Metabolomics Standards Initiative (MSI), metabolites confirmed using authentic standards with concordant RT and MS/MS spectra were classified as MSI Level 1. Metabolites annotated by MS/MS spectral matching to the in-house or public spectral libraries, but without direct confirmation by authentic standards in the analyzed samples, were classified as MSI Level 2. Annotations supported only by in silico prediction or MetDNA-based propagation were not considered high-confidence Level 1/2 identifications and were interpreted conservatively.

For differential metabolite analysis, significantly altered metabolites were defined as those with variable importance in projection (VIP) > 1 from OPLS-DA and *p* < 0.05 (Student’s *t* test). Data were log_2_-transformed and mean-centered before OPLS-DA, and model overfitting was evaluated using 200 permutation tests.

**RNA extraction and transcriptome sequencing**

Total RNA was extracted from small intestinal tissues using TRIzol reagent according to standard protocols. RNA concentration and integrity were assessed using a NanoDrop spectrophotometer (Thermo Fisher Scientific, USA) and an Agilent 2100 Bioanalyzer (Agilent Technologies, USA). High-quality RNA was reverse-transcribed into cDNA, and sequencing libraries were constructed according to the manufacturer’s instructions. Paired-end sequencing (PE150) was performed on the Illumina platform.

**DATA PROCESSING AND STATISTICAL ANALYSIS**

**16S rRNA Sequencing Analysis**

Raw 16S rRNA sequencing data generated on the Illumina NovaSeq platform were first processed using fastp (v0.22.0) for quality control and merged reads. Chimeric sequences were removed with Vsearch (v2.22.1) to obtain high-quality effective tags. Amplicon sequence variants (ASVs) were inferred through denoising, and rarefaction curves and data normalization were performed in R (v4.2.0). Alpha- and beta-diversity were assessed using USEARCH (v10.0.240). Taxonomic annotation was conducted with the SINTAX algorithm in Vsearch against the ribosomal database project (RDP) reference database (confidence threshold 0.8). Differential abundance analysis was performed using edgeR (*p* < 0.05, FDR < 0.2). Taxa with significant group differences were identified using LEfSe, and microbial functional prediction was conducted using PICRUSt2 with KEGG annotations. Differential pathways were determined by LinDA (*p* < 0.05). Phylogenetic trees were visualized using iTOL (v7.0).

**Microbiome age modeling and age-matched compositional deviation**

A WT-referenced microbiome age model was constructed from ASV count tables generated from fecal 16S rRNA gene sequencing data using WT samples only, such that WT animals defined the reference developmental trajectory of the gut microbiome. ASVs detected in fewer than 10% of WT samples were excluded. The remaining counts were converted to relative abundances after addition of a pseudocount of 0.5 and then subjected to centered log-ratio (CLR) transformation. A regularized regression model was implemented using the glmnet package in R. The elastic-net mixing parameter (alpha) was selected from the grid 0, 0.25, 0.5, 0.75, and 1 based on cross-validated mean squared error in WT samples. Model performance was then evaluated in WT animals using leave-one-animal-out validation, in which all longitudinal samples from one WT monkey were withheld together in each fold. Performance was summarized by the Pearson correlation coefficient (*r*), root mean squared error (RMSE), and mean absolute error (MAE) between chronological age and predicted microbiome age in WT samples. The final WT-derived model was subsequently applied to all WT and RTT samples to generate predicted microbiome age values. Predicted microbiome age was interpreted as a WT-referenced microbiome maturity score rather than an exact estimate of chronological age.

To quantify divergence from the expected WT microbiome configuration at each age, age-matched compositional deviation was calculated as the Euclidean distance in CLR-transformed ASV space between each sample and the centroid of WT samples at the same chronological age. Group differences between WT and RTT samples were assessed within each age stratum using two-sided Wilcoxon rank-sum tests.

**Machine Learning Analysis**

**Data preprocessing and feature construction**

Machine learning analysis was performed based on genus-level abundance tables generated from statistical analysis of metagenomic profiles (STAMP). Taxonomic annotations were first parsed to extract hierarchical levels from phylum to genus, and unannotated taxa were removed. Raw abundance data were then converted to relative abundances and log_2_(x+1)-transformed to reduce the dominance of highly abundant taxa and stabilize variance. Subsequently, the feature matrix was standardized using Z-score normalization to eliminate scale differences among genera.

The external validation dataset was constructed from a public dataset. Briefly, the OTU table was first aggregated to the genus level, and unclassified taxa as well as low-quality samples with total reads < 100 were removed. Relative abundance transformation, log_2_(x+1)-transformed, and Z-score normalization were then performed. To ensure consistency in feature dimensions between the internal and external datasets, only shared genus-level features were retained for downstream modeling. All feature matrices were organized in a sample-by-genus format, and phenotype labels were encoded as RTT = 1 and WT = 0.

**Data splitting and validation strategy**

The internal cohort included 18 animals (9 RTT and 9 WT), yielding a total of 72 samples. After data normalization, all samples were divided into training and test sets at a ratio of 70% and 30%, respectively, using stratified sampling to preserve class distribution as much as possible. During model development, 10-fold cross-validation was performed exclusively within the training set for model training, model selection, and hyperparameter tuning. The test set was not involved in model fitting or parameter optimization. An external public dataset was used as an independent validation cohort to further evaluate model generalizability across different data sources.

Given that the internal dataset was derived from a longitudinal design, samples collected from different time points in the same animal may not be fully independent. Therefore, sample-level splitting of the training and test sets may introduce a certain degree of information sharing. Accordingly, the machine learning analysis was primarily intended as an exploratory approach to evaluate the discriminatory potential of microbial features for RTT status, and the results will require further validation in larger datasets with independent subject-level partitioning.

**Feature selection**

To reduce redundancy and noise in high-dimensional microbiome features, Lasso regression was first applied for feature selection. This method incorporates L1 regularization within a logistic regression framework, and the optimal penalty parameter (λ) was determined by cross-validation. Genera with non-zero coefficients were retained as candidate features for downstream model construction.

**Neural network model construction**

The neural network classifier was implemented as a multilayer perceptron (MLP). The model consisted of fully connected layers with 16, 8, and 4 neurons in the hidden layers, respectively. ReLU activation functions were used in all hidden layers, and a sigmoid activation function was applied in the output layer to generate binary classification probabilities. To reduce overfitting, L2 regularization and dropout were introduced in the hidden layers. Model training was performed using the Adam optimizer with binary cross-entropy loss. The main hyperparameters included hidden-layer architecture, learning rate, batch size, number of training epochs, dropout rate, and L2 regularization strength, all of which were optimized within the training set using cross-validation.

**Other machine learning models**

In addition to the MLP model, XGBoost, gradient boosting machine (GBM), logistic regression, stepwise logistic regression, ridge regression, elastic net regression, linear discriminant analysis, quadratic discriminant analysis, k-nearest neighbors, decision tree, random forest, naive Bayes, and support vector machine models were also constructed. For XGBoost and GBM, key parameters including learning rate, tree depth, number of iterations/trees, and interaction depth were optimized using cross-validation combined with grid search implemented in the caret package. For support vector machines, linear, polynomial, and radial basis function kernels were evaluated. Ridge regression and elastic net models were optimized using cv.glmnet. In some analyses, hybrid models were further constructed using Lasso-selected features as input to reduce dimensionality and improve model stability.

**Handling of class imbalance**

Class distribution was assessed before model training. Because the internal cohort contained equal numbers of RTT and WT animals (9 per group), the overall class distribution was balanced. Therefore, no additional class imbalance correction methods, including oversampling, undersampling, SMOTE, or class-weight adjustment, were applied. All models were trained and evaluated using the original class distribution.

**Model evaluation**

Model performance was assessed using accuracy, recall, F1 score, and area under the receiver operating characteristic curve (AUC). For models producing continuous probability outputs, predicted probabilities were converted into binary labels based on predefined thresholds, and performance metrics were calculated in the training set, internal test set, and external validation cohort. To compare the overall performance of different models, performance metrics across datasets were summarized, and genera that showed stable importance across multiple models were further prioritized as candidate microbial biomarkers.

**Mendelian Randomization Analysis**

A two-sample Mendelian randomization (TSMR) framework was applied to evaluate the potential causal associations between gut microbial features and cardiovascular-related phenotypes. Exposure data were obtained from summary statistics in the MiBioGen consortium, including 119 gut microbial genera. Single-nucleotide polymorphisms (SNPs) associated with microbial abundance were selected as instrumental variables using the following criteria: *p* < 1×10^-5^, linkage disequilibrium clumping threshold of *r^2^* < 0.001 with a physical distance > 10,000 kb, and minor allele frequency (MAF) > 1%, to maximize the independence of the selected instruments.

Outcome data were obtained from the IEU OpenGWAS database. The cardiovascular-related phenotypes included dilated cardiomyopathy, ventricular arrhythmia, cardiac dysrhythmias, fetal heart rate abnormality, resting heart rate, electrocardiogram (ECG) heart rate, and heart rate variability. Exposure and outcome datasets were first harmonized using the harmonise_data function to align effect alleles, and palindromic SNPs with ambiguous strand orientation were removed. Potential outlier instruments were then identified using the MR-PRESSO global test and were iteratively removed when necessary to improve the robustness of the estimates.

Causal effect estimation was primarily performed using the inverse-variance weighted (IVW) method. Weighted median, MR-Egger regression, weighted mode, and simple mode methods were additionally applied as complementary analyses to assess the consistency of the results under different model assumptions. Directional pleiotropy was evaluated using the MR-Egger intercept test, and heterogeneity among instrumental variables was assessed using Cochran’s Q statistic. When significant heterogeneity was detected, leave-one-out analysis was further conducted to evaluate the influence of individual SNPs on the overall estimates.

Effect estimates for each microbial genus and cardiovascular-related phenotype were reported as odds ratios (ORs) with corresponding 95% confidence intervals and visualized using forest plots. Given the biological complexity of genetic instruments related to gut microbiota, these analyses were primarily intended to indicate potential associations and their directions, thereby providing clues for subsequent mechanistic studies and further validation.

**SCFAs Data Analysis**

After batch-effect correction using ComBat, differences in the seven quantified SCFAs between RTT and WT monkeys at each time point were assessed using Wilcoxon rank-sum tests. Associations between microbial/SCFAs modules and WT/RTT age groups were evaluated by Spearman correlations analysis. Multivariate analysis was performed using Orthogonal partial least-squares discriminant analysis (OPLS-DA) (R²Y = 0.993, Q² = 0.667, *p* < 0.005), with differential metabolites defined as VIP > 1, *p* < 0.05, and |log₂FC| > 1. Multiple-testing correction was not performed; therefore, the corresponding findings should be interpreted as exploratory results.

**Metabolomic Data Analysis**

Raw metabolomic data were processed with Analyst (v1.6.3) for peak extraction. Metabolites were identified using the HMDB, MassBank, Metlin, and in-house standards. Differential metabolites were determined using OPLS-DA and Student's t-test with thresholds of VIP > 1, *p* < 0.05, and |log₂FC| > 1. Pathway enrichment analysis was performed in MetaboAnalyst (v6.0) *(Impact* > 0.1*, p <* 0.01). A metabolite-gene-disease interaction network and joint pathway analysis were constructed to uncover regulatory mechanisms underlying metabolic alterations. Multiple-testing correction was not performed; therefore, the corresponding findings should be interpreted as exploratory results.

**RNA-seq Data Analysis**

RNA-seq data were subjected to quality control using FastQC (v0.11.9) to remove low-quality reads and adaptor sequences. Reads were aligned to the monkey reference genome (Macaca_mulatta.Mmul_10.107.gtf) using HISAT2 (v2.2.1), followed by transcript assembly and quantification using StringTie (v2.2.1). Differential gene expression analysis was conducted using DESeq2 with rlog normalization, and significant differentially expressed genes (DEGs) were defined by padj < 0.05 and |log₂FC| > 1. Gene set enrichment analysis (GSEA) was performed using the GSEA function in the clusterProfiler package (v4.6.2). KEGG pathway annotations for Macaca mulatta were used as the reference gene sets. Enrichment plots were generated using gseaplot2 from the enrichplot package (v1.18.4). Core enrichment genes were extracted from the GSEA results.

**Supplementary Figures**


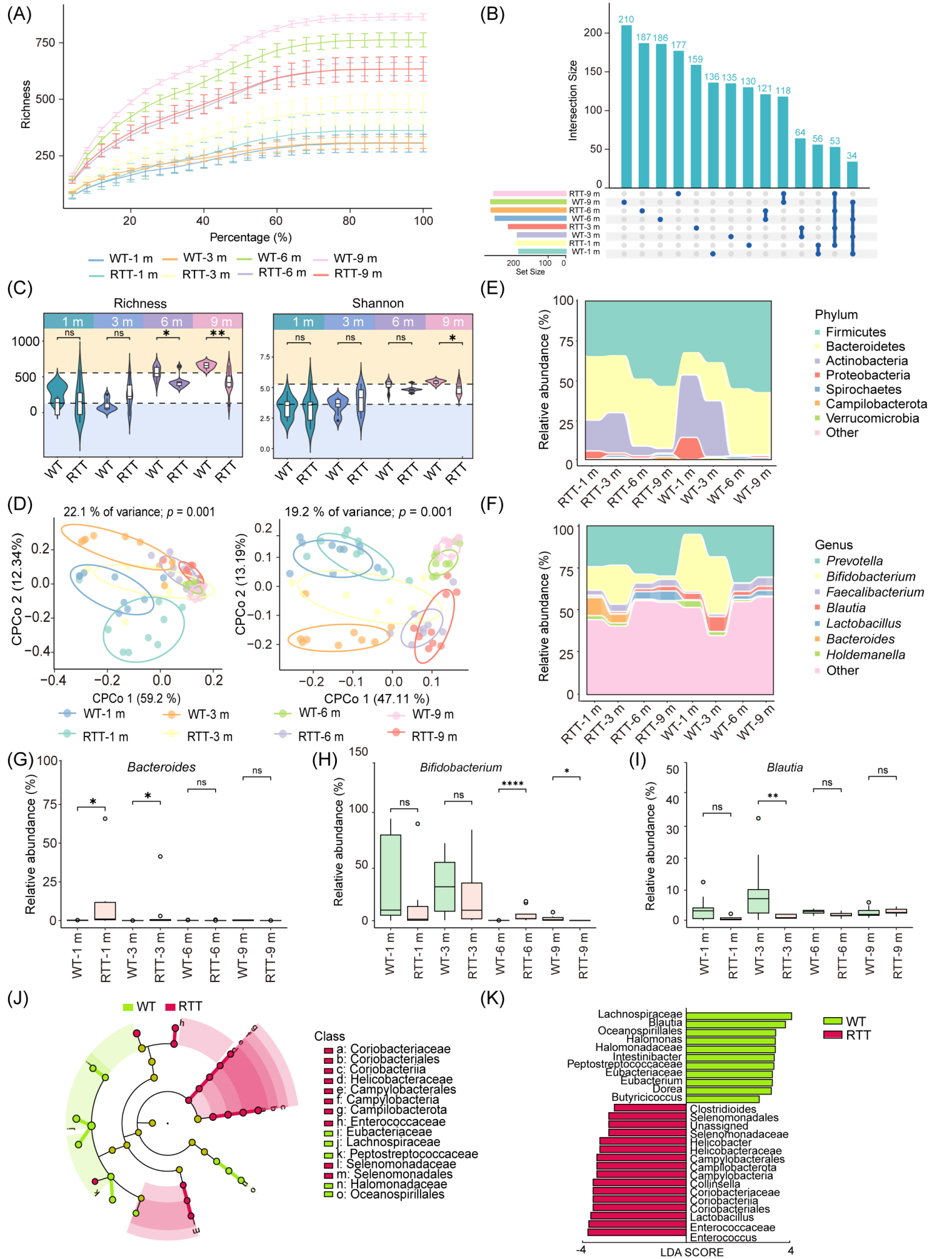


**Figure S1 Longitudinal profiling of gut microbial diversity and community structure in Rett syndrome (RTT) monkeys. (A) Species accumulation curves showing the increase in observed richness with sampling percentage in wild-type (WT) and RTT monkeys at 1, 3, 6, and 9 months of age. (B) UpSet plot showing the numbers of shared and group-specific amplicon sequence variants (ASVs) across WT and RTT samples at different developmental stages. (C) Alpha diversity of the gut microbiota assessed by richness (left) and Shannon index (right) in WT and RTT monkeys across development. (D)** Beta diversity analysis based on Bray-Curtis (left) and UniFrac (right) distances, showing separation between WT and RTT monkeys **across developmental stages. (E, F) Relative abundances of major bacterial phyla (E) and genera (F) in WT and RTT monkeys at 1, 3, 6, and 9 months of age. (G-I) Relative abundances of *Bacteroides* (G), *Bifidobacterium* (H), and *Blautia* (I) in WT and RTT monkeys across development. (J, K) Differentially enriched taxa identified by LEfSe analysis, shown as a cladogram (J) and corresponding LDA score plot (K).** n = 9 independent biological replicates per group. Statistical significance was assessed using unpaired two-tailed *t*-tests. ****p* < 0.05; ***p* < 0.01; ****p* < 0.001**; ns, not significant.


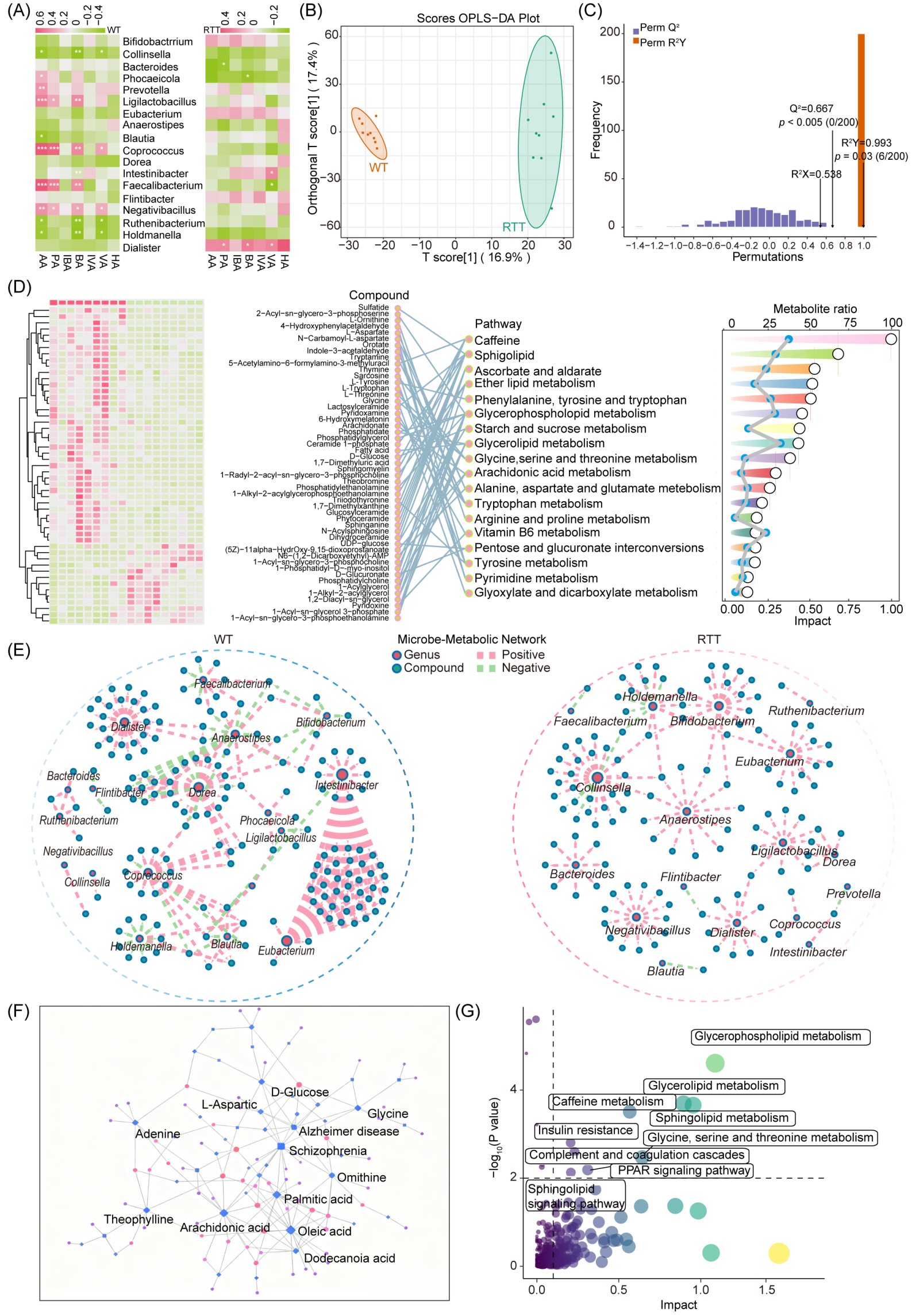


**Figure S2 Fecal metabolomic alterations and microbiota-metabolite network remodeling in RTT monkeys.**

(A) Pearson correlation heatmaps showing associations between the 18 RTT-associated microbial genera and seven fecal short-chain fatty acids (SCFAs) in WT and RTT monkeys. RTT monkeys exhibited globally weakened correlations together with partial reversal of association patterns. (B) Orthogonal partial least-squares discriminant analysis (OPLS-DA) showing separation between WT and RTT fecal metabolomic profiles at 9 months of age. (C) Permutation test of the OPLS-DA model, showing the distributions of permuted R²Y and Q² values and supporting model robustness. (D) Heatmap of differentially abundant fecal metabolites and their associated enriched metabolic pathways in WT and RTT monkeys at 9 months of age. (E) Microbiota-metabolite interaction networks constructed from differential metabolites and 18 RTT-associated microbial genera in WT and RTT monkeys, showing reduced network complexity and altered centrality in RTT. (F) Integrated metabolite-gene interaction network highlighting highly connected molecular hubs, including D-glucose, glycine, oleic acid, palmitic acid, L-aspartic acid, adenine, theophylline, and ornithine. (G) Joint pathway analysis of differentially abundant metabolites and differentially expressed genes, showing significant alterations in glycerophospholipid metabolism, glycerolipid metabolism, sphingolipid metabolism, and the peroxisome proliferator-activated receptor (PPAR) signaling pathway in RTT monkeys. For panels A-E, n = 9 biologically independent monkeys per group.


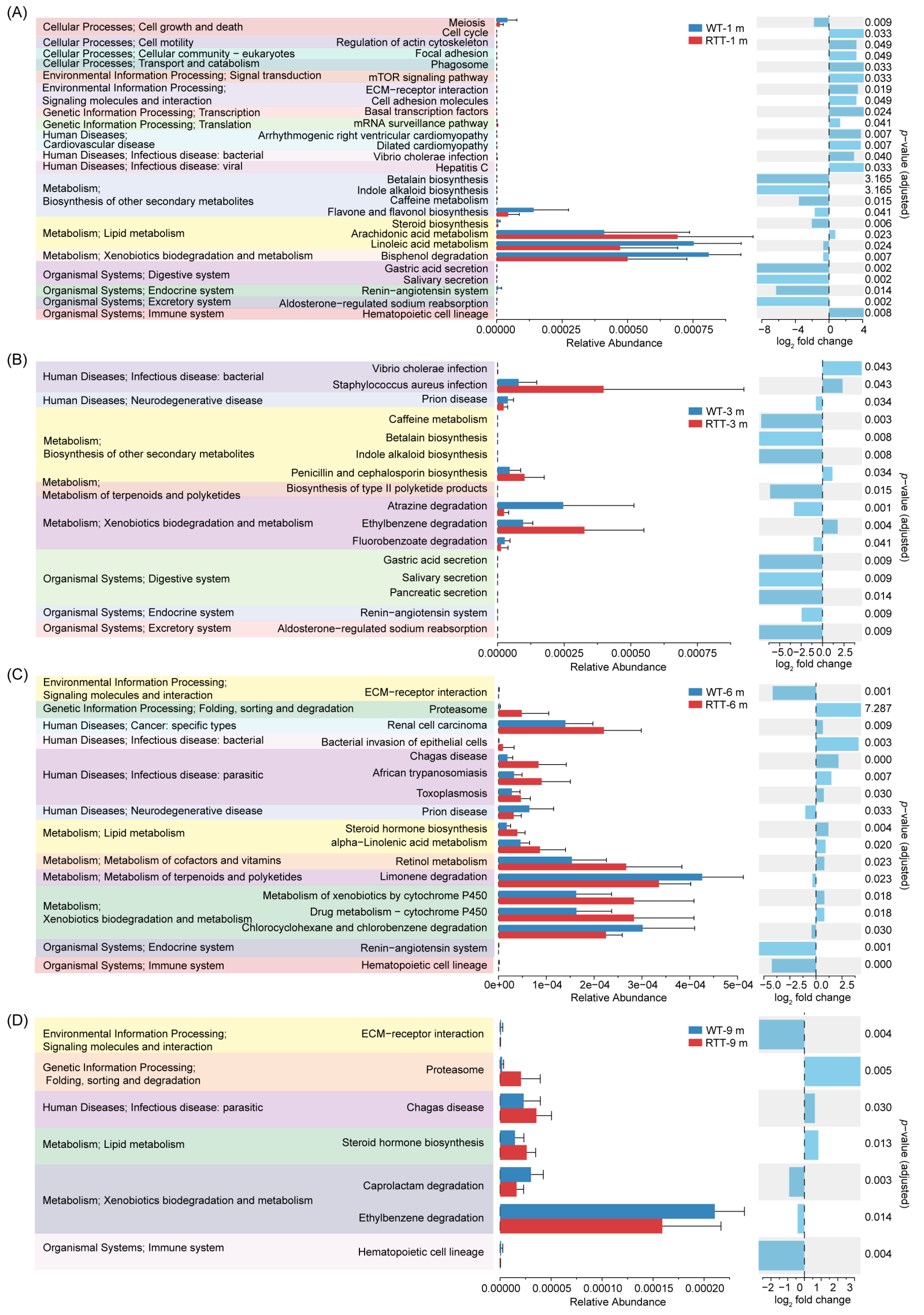


**Figure S3 Altered predicted microbial functional potential in RTT monkeys across development. (A-D) Predicted functional profiles of gut microbiota in WT and RTT monkeys at 1 (A), 3 (B), 6 (C), and 9 (D) months of age, inferred using PICRUSt2 based on 16S rRNA gene sequencing data and Kyoto Encyclopedia of Genes and Genomes (KEGG) pathway annotation. RTT monkeys showed a relative reduction in metabolic functions together with enrichment of gene families mapped to KEGG categories annotated as inflammation- and cardiomyopath-related. These annotations represent collections of conserved gene modules involved in fundamental biological processes rather than evidence of specific infectious or cardiac disease states. For panel A-D, n = 9** independent biological replicates per group at each time point.

**
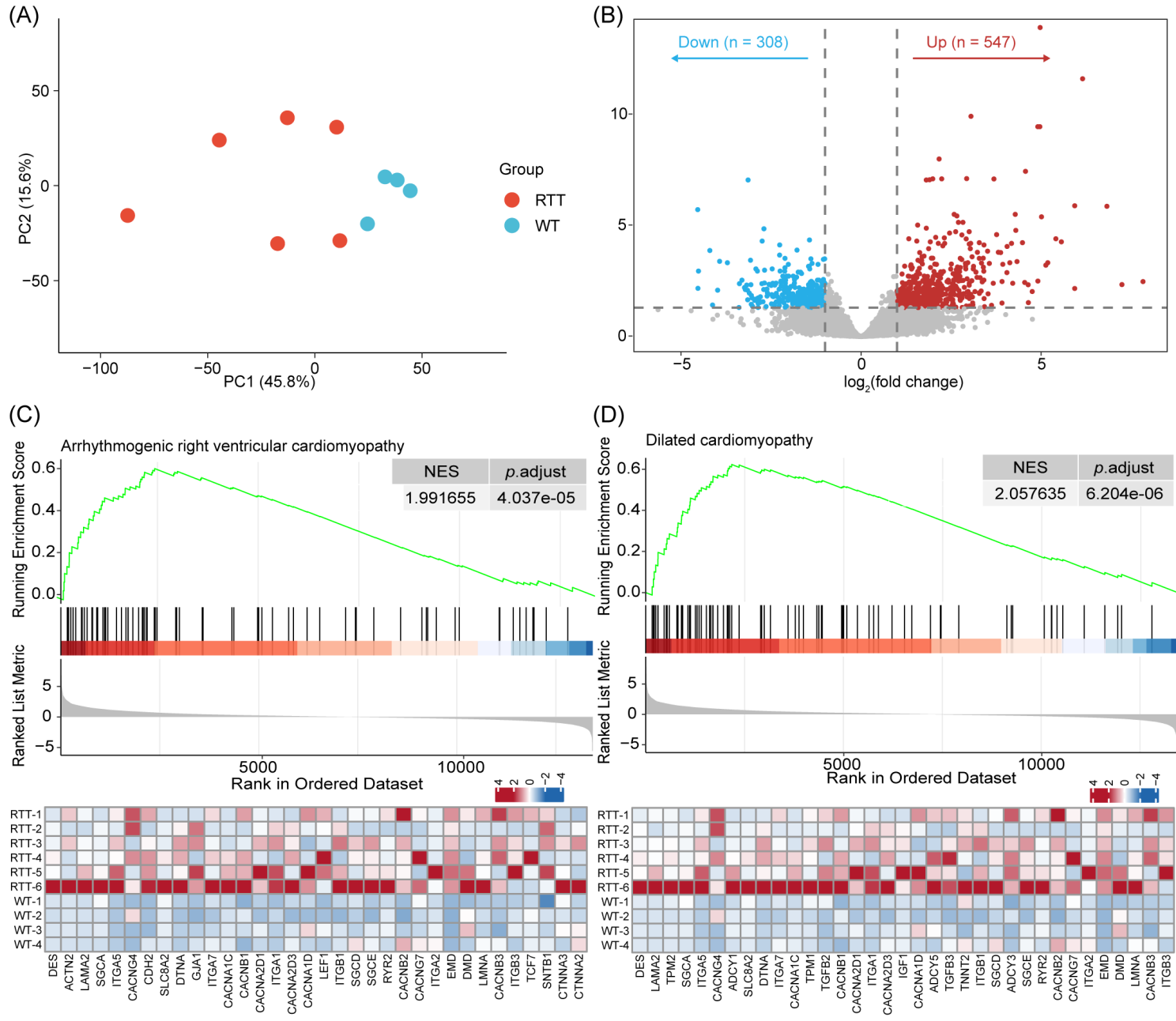
**

**Figure S4 Transcriptomic alterations in small intestinal tissues from WT and RTT monkeys. (A) Principal component analysis (PCA) of small intestinal transcriptomes from WT and RTT monkeys. (B) Volcano plot showing differentially expressed genes between RTT and WT groups. Genes upregulated in RTT relative to WT are shown in red, and genes downregulated in RTT relative to WT are shown in blue. (C-D) Gene set enrichment analysis (GSEA) of KEGG gene sets corresponding to arrhythmogenic right ventricular cardiomyopathy (ARVC) (C) and dilated cardiomyopathy (DCM) (D). Genes were ranked by log_2_ fold change for RTT versus WT, and positive enrichment indicates enrichment among genes upregulated in RTT. Heatmaps below each GSEA plot show the scaled expression of the corresponding core enrichment genes across WT and RTT samples. The cardiomyopathy-related annotations should be interpreted as exploratory pathway-level signals rather than evidence of overt cardiac pathology. n** = 6 independent biological replicates for the RTT group and n = 4 independent biological replicates for the WT group.


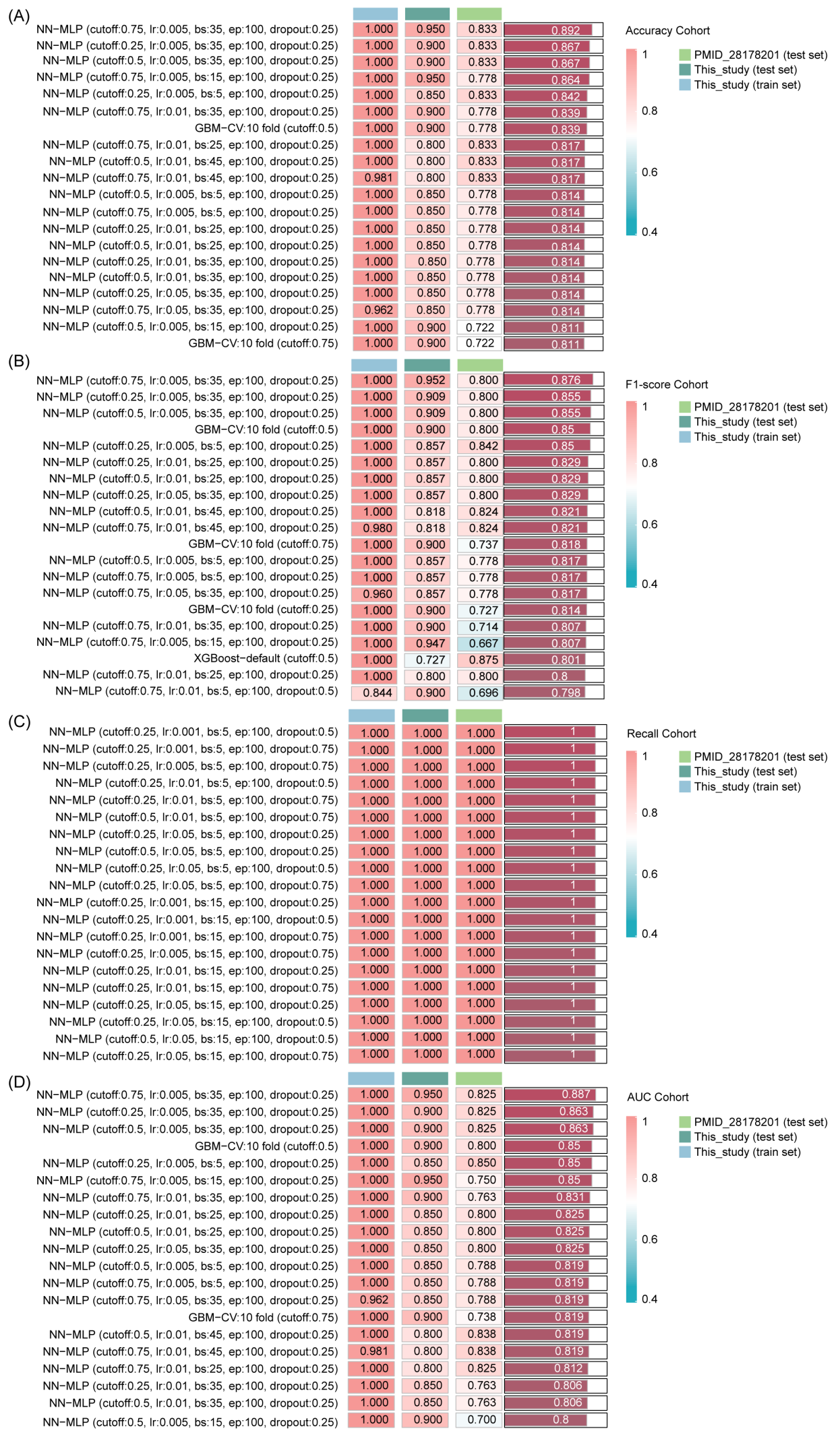


**Figure S5 Performance evaluation of machine learning models for identifying RTT associated microbial genera.** Comparative performance of the top 20 models selected from 279 model combinations generated using 15 machine-learning algorithms, evaluated by accuracy (A), F1 score (B), recall (C), and area under the receiver operating characteristic curve (AUC) (D).

**
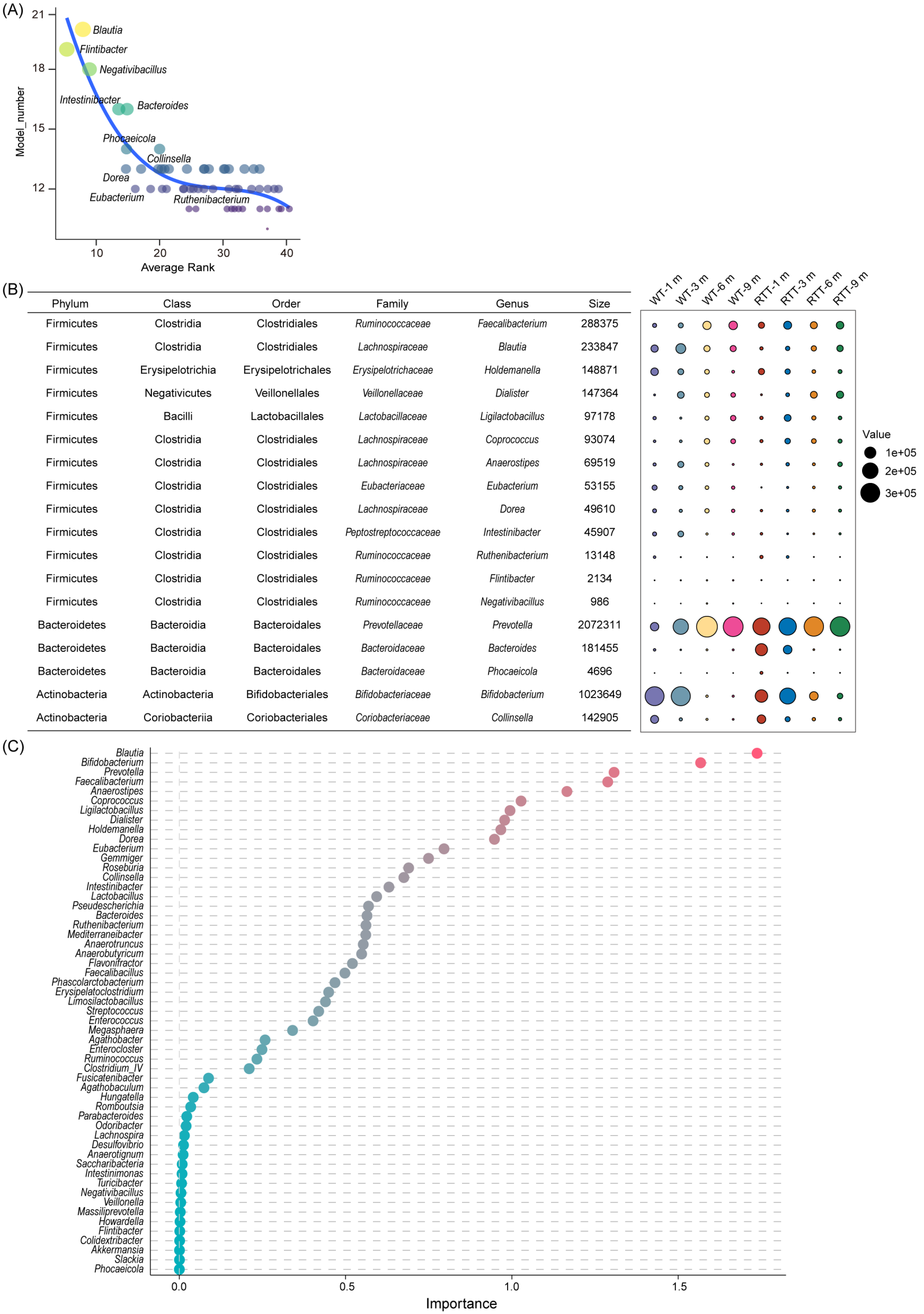
**

**Figure S6** **Prioritization of RTT-associated microbial features by machine-learning analysis. (A) Summary of feature importance across 279 model combinations, identifying the top 10 genera recurrently prioritized across algorithms. (B) Eighteen RTT-associated microbial genera identified by integrating consensus feature aggregation and neural network-based ranking, together with their taxonomic assignments and relative abundances across developmental stages. (C) Genus-level feature importance derived from the neural network-based multilayer perceptron (NN-MLP) model.**


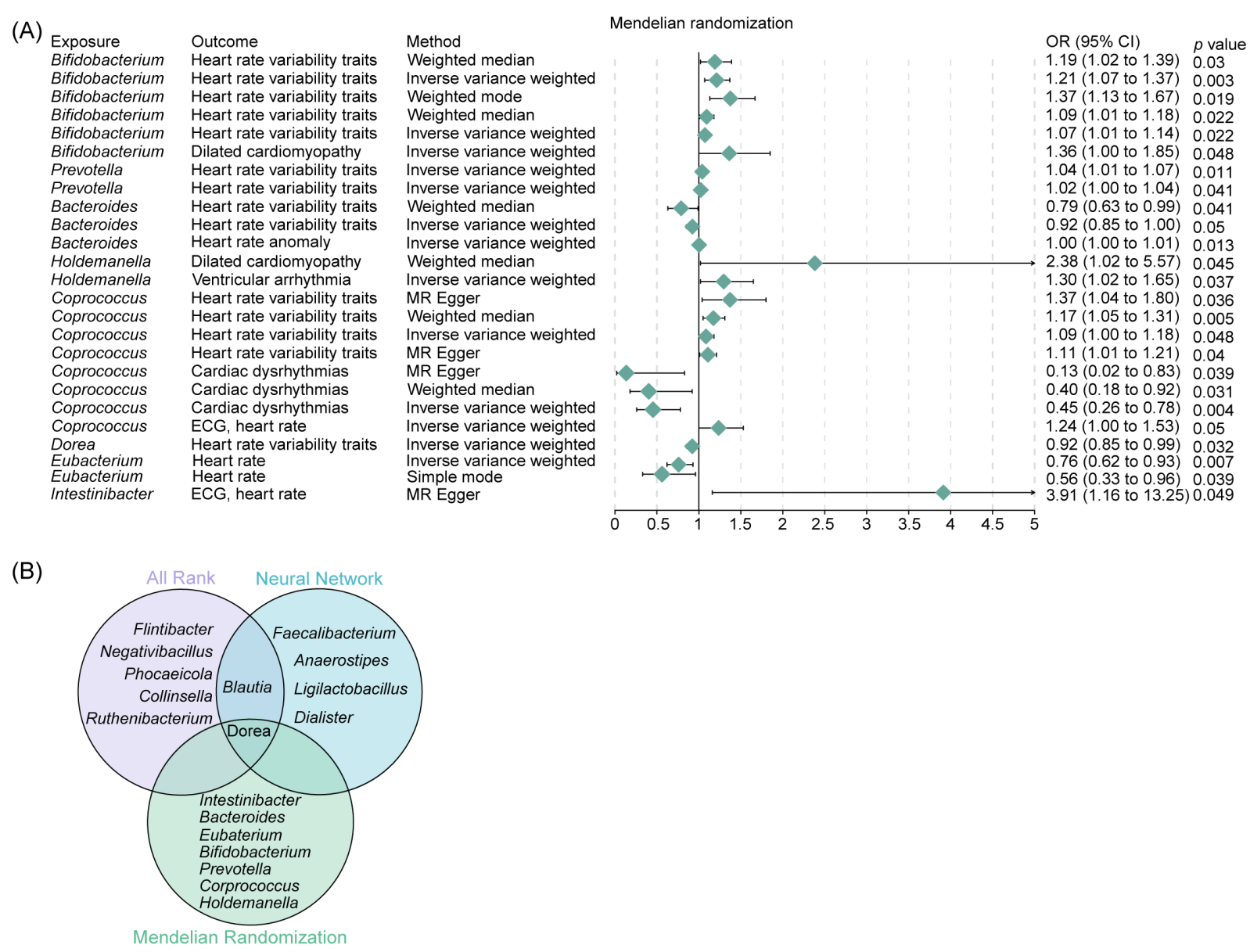


**Figure S7 Exploratory Mendelian randomization analysis of RTT-associated microbial genera and cardiovascular-related traits. (A) Two-sample Mendelian randomization analysis evaluating nominal associations between RTT-associated genera and cardiovascular-related traits, including arrhythmia- and cardiomyopathy-related phenotypes. These findings should be interpreted as hypothesis-generating rather than causal. (B) Venn diagram showing the overlap among microbial genera prioritized by consensus ranking, neural network-based analysis, and Mendelian randomization, with *Dorea* identified as a shared feature across analytical approaches.**
